# Orbital *SOX10*-mutant schwannoma with plexiform growth: Expanding the histopathological spectrum of a new molecular group

**DOI:** 10.1093/jnen/nlad080

**Published:** 2023-10-13

**Authors:** Ansar A Wali, Robin Yang, Shannath L Merbs, Fausto J Rodriguez, Charles G Eberhart, Calixto-Hope G Lucas

**Affiliations:** Department of Pathology, Johns Hopkins University School of Medicine, Baltimore, Maryland, USA; Department of Plastic and Reconstructive Surgery, Johns Hopkins University School of Medicine, Baltimore, Maryland, USA; Department of Ophthalmology and Visual Sciences, University of Maryland School of Medicine, Baltimore, Maryland, USA; Department of Pathology, University of California Los Angeles School of Medicine, Los Angeles, California, USA; Department of Pathology, Johns Hopkins University School of Medicine, Baltimore, Maryland, USA; Department of Pathology, Johns Hopkins University School of Medicine, Baltimore, Maryland, USA

To the Editor:

While *NF2*, *LZTR1*, and *SMARCB1* mutations along with *HTRA1* gene fusions are well-recognized oncogenic drivers of schwannoma development (1), a recent report revealed nearly one-third of sporadic schwannomas harbor *SOX10* in-frame insertion/deletion mutations (2). Interestingly, these *SOX10*-mutant schwannomas involved the facial, trigeminal, and vagus nerves rather than the vestibulocochlear nerve. Here, we described an orbital plexiform schwannoma harboring a novel *SOX10* 6 base pair in-frame insertion, further extending the clinicopathologic spectrum of recently described *SOX10*-mutant schwannomas.

**Figure 1:**
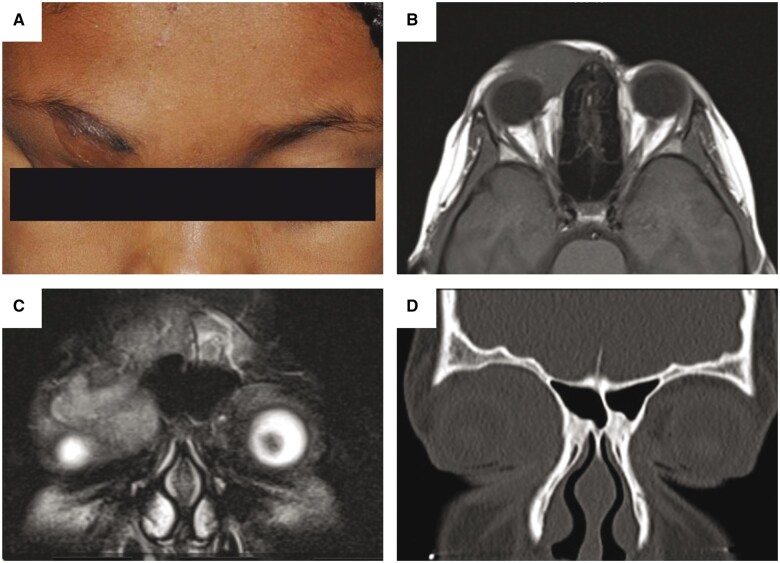
Clinical and radiographic features. A subcutaneous mass involving the superior nasal right periorbital region was noted on physical exam **(A)**. Magnetic resonance imaging demonstrated the mass was primarily T1 isointense **(B)** and heterogeneously T2 minimally hypointense **(C)**. Computerized tomography imaging demonstrated remodeling of the anterior wall of the right frontal sinus and orbital roof **(D).**

An 8-year-old girl presented with a slowly growing lesion of the right upper brow that had gradually enlarged over 4 years. She was otherwise healthy and had no familial history of tumors. Physical exam revealed a subcutaneous, soft, painless mass involving the superior nasal right periorbital region (Fig. 1A). Ultrasonography demonstrated a heterogeneous lesion approximately measuring 2.2 × 1.9 × 1.3 cm with no internal vascularity noted. Magnetic resonance imaging demonstrated a right preseptal mass extending into the superior-nasal orbit that was primarily T1 isointense (Fig. 1B) and heterogeneously T2 minimally hypointense (Fig. 1C) with increased internal enhancement. Computerized tomography scan showed remodeling of the anterior wall of the right frontal sinus and orbital roof (Fig. 1D). She underwent biopsy of the tumor followed by a definitive resection. Intraoperatively, a firm, tan-white tumor was identified. A neurovascular branch from the supraorbital nerve was observed entering the lesion.

**Figure 2:**
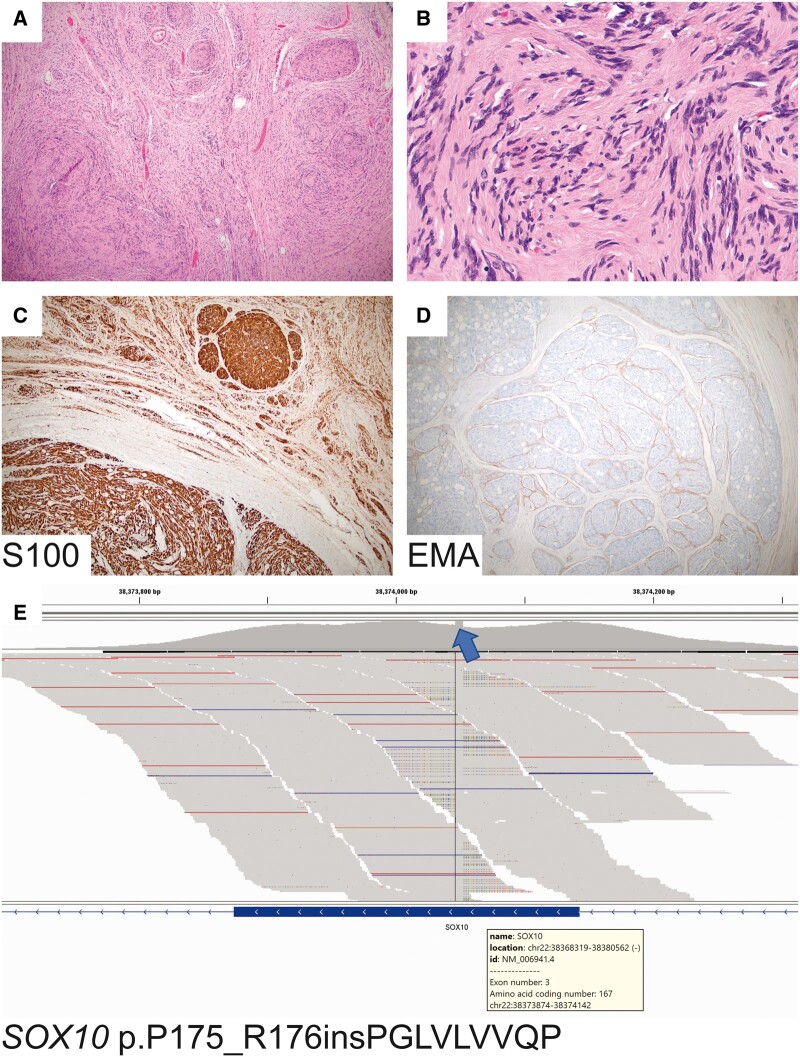
Morphologic, immunohistochemical, and molecular features. Sections reveal a solid tumor composed of spindled cells arranged in multiple nodules of varying sizes with intervening bands of fibrous tissue **(A)**. In some areas, tumor nuclei were arranged in palisades **(B)**. The neoplastic cells were diffusely immunoreactive for S100 **(C)**. Epithelial membrane antigen (EMA) highlighted the perineurium surrounding tumor nodules **(D)**. Next-generation sequencing revealed a *SOX10* p.P175_R176insPGLVLVVQP 6 base pair in-frame insertion within exon 3 **(E).**

Histologic sections revealed a solid neoplasm composed of spindled cells with wavy nuclei and variable architecture, including plexiform areas (Fig. 2A), sheetlike arrangements, and vague nuclear palisading (Fig. 2B). The neoplastic cells expressed SOX10 and S100 (Fig. 2C). Neurofilament protein immunostain highlighted twigs of nerve at the periphery but intratumoral axons were absent. Epithelial membrane antigen highlighted perineurium (Fig. 2D). CD34 labeled blood vessels and scattered stromal cells. INI1 expression was intact. Next-generation DNA sequencing studies detected a *SOX10* p.P175_R176insPGLVLVVQP mutation with a variant allele frequency of 31% (RefSeq NM_006941, Fig. 2E). Relative gain of chromosome 11 was also noted. No other pathogenic alterations were identified. Notably, *NF2*, *LZTR1*, *SMARCB1*, and chromosome 22q were intact.

While the *SOX10* p.P175_R176insPGLVLVVQP mutation identified in this current case is novel, the position overlaps with previously reported in-frame insertion/deletion mutations clustered at the C-terminus of the high-mobility group-box domain (2). Schwannomas harboring *SOX10* mutations predominantly arise from non-vestibular cranial nerves, whereas schwannomas arising along the vestibulocochlear nerve (cranial nerve VIII) typically harbored *NF2* mutations. In line with this observed anatomic enrichment, this case demonstrates involvement of the supraorbital nerve, a distal branch of the trigeminal nerve (cranial nerve V). Thus, the integrated diagnosis in this case was “plexiform schwannoma, *SOX10*-mutant.”

Notably, plexiform architecture was not mentioned in the recent report of *SOX10*-mutant schwannomas, so this case further extends the morphologic spectrum of this biologic subgroup of tumors. Plexiform schwannomas are rare but arise predominantly in the superficial soft tissues of the head and neck or trunk areas (3, 4). Deep-seated lesions are also encountered (5). Histologically, they are defined by a multinodular growth pattern typically lacking a well-defined external capsule (6). They can represent surgically challenging cases with a predilection for local progression or recurrence (3, 7). Whether other plexiform schwannomas of these regions harbor *SOX10* mutations remains to be explored. Notably, most plexiform schwannomas are sporadic although rare cases arise in patients with tumor predisposition syndromes (8). To our knowledge, oncogenic drivers in sporadic plexiform schwannomas have not been described. Thus, we speculate that *SOX10* mutations may contribute to plexiform growth in a subset of schwannomas, but additional cases are needed to explore this possibility. It will also be important to determine optimal therapies for these histologic and molecular schwannoma variants.
